# SPR Biosensors in Direct Molecular Fishing: Implications for Protein Interactomics

**DOI:** 10.3390/s18051616

**Published:** 2018-05-18

**Authors:** Anna Florinskaya, Pavel Ershov, Yuri Mezentsev, Leonid Kaluzhskiy, Evgeniy Yablokov, Alexei Medvedev, Alexis Ivanov

**Affiliations:** Institute of Biomedical Chemistry, 119121 Moscow, Russia; aflorin@bk.ru (A.F.); pavel79@inbox.ru (P.E.); la-kaluzhskiy@yandex.ru (L.K.); evgeyablokov1988@mail.ru (E.Y.); professor57@yandex.ru (A.M.); alexei.ivanov@ibmc.msk.ru (A.I.)

**Keywords:** SPR biosensors, direct molecular fishing, protein interactomics, size exclusion chromatography (SEC), LC-MS/MS protein identification, isatin

## Abstract

We have developed an original experimental approach based on the use of surface plasmon resonance (SPR) biosensors, applicable for investigation of potential partners involved in protein–protein interactions (PPI) as well as protein–peptide or protein–small molecule interactions. It is based on combining a SPR biosensor, size exclusion chromatography (SEC), mass spectrometric identification of proteins (LC-MS/MS) and direct molecular fishing employing principles of affinity chromatography for isolation of potential partner proteins from the total lysate of biological samples using immobilized target proteins (or small non-peptide compounds) as ligands. Applicability of this approach has been demonstrated within the frame of the Human Proteome Project (HPP) and PPI regulation by a small non-peptide biologically active compound, isatin.

## 1. Introduction

There is increasing evidence that in living systems proteins exist and function within stable or dynamic molecular complexes [1]. Protein–protein interactions (PPIs) determining formation and lifespan of such complexes attract much interest; they are extensively studied by using various bioinformatic, genomic, and biochemical technologies [2,3,4]. In this context, biochemical methods are the most reliable ones: using these methods researchers investigate PPIs under conditions close to physiological.

Biochemical methods employ the strategy of molecular fishing for isolation of protein complexes and subsequent mass spectrometry identification of potential protein partners.

Molecular fishing is a variant of affinity-based isolation of target proteins from a lysate of the biological material due to specific interaction between the immobilized ligand (a bait molecule) and its putative (one or several) functionally competent partners (pray molecules) [4,5,6]. Various compounds have been used as the bait molecules; these include small organic molecules [7,8], proteins and nucleic acids [4].

In this review, we have summarized results of our studies on the use of SPR-based approach for direct molecular fishing of proteins from lysates of biological materials and identification of prey proteins by mass spectrometry. We initially consider a general strategy for the use of SPR biosensors at the particular experimental stages of molecular fishing with special attention to the SPR-based optimization of experimental protocols (including immobilization of bait proteins on a carrier, evaluation of intactness of the immobilized bait protein, and optimization of protocols for preparation of tissue/cell culture lysates, etc.). After that we consider the role of the SPR biosensor technology in the SPR-based analytical fishing. Finally, we demonstrate applicability of the SPR biosensor technology for analysis of ligand protein interactions using non-peptide small molecules as baits.

## 2. Direct Molecular Fishing

**Figure 1:**
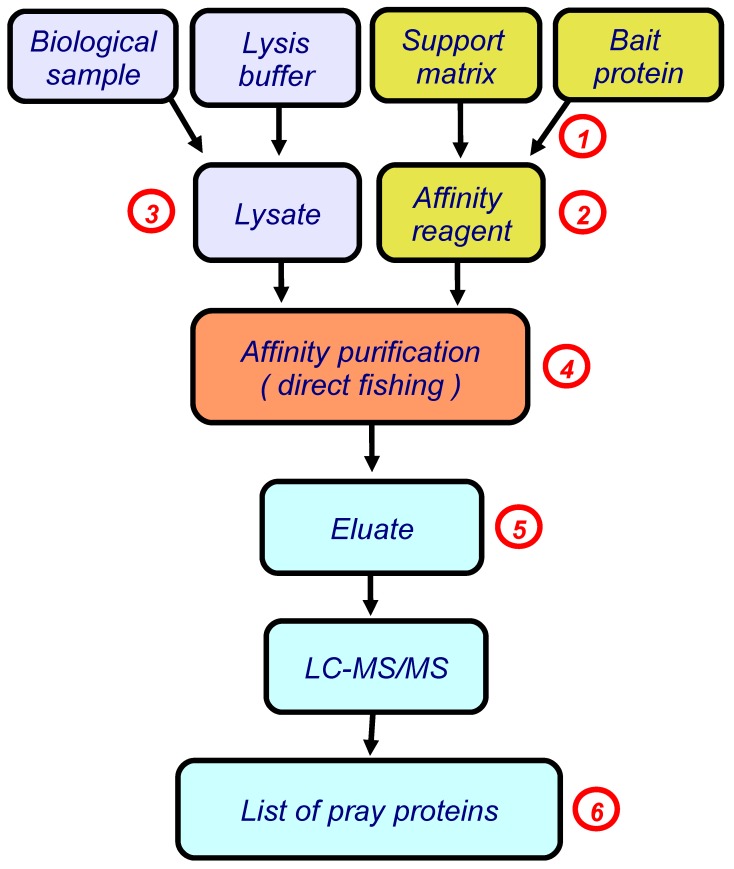
The block scheme illustrating direct molecular fishing of potential partners of protein–protein interactions. Red numbers indicate the steps in which a SPR biosensor could be used (details are given in the text, numbers in red circles correspond to points considered in Section 3).

In our studies, we use the simplest version of this approach, direct molecular fishing, in which the target protein (a bait protein) is immobilized on the sorbent, as an affinity ligand. Figure 1 shows a basic block scheme illustrating this approach.

Applicability of direct molecular fishing for protein interactomics has been demonstrated in model experiments with isolation of a known partner protein in the presence of proteins with a high level of nonspecific sorption [9,10]. All the quantitative interaction analysis of the target partner proteins and nonspecific proteins (noise) was performed using the SPR biosensor Biacore 3000 (GE Healthcare, Chicago, IL, USA). We used paramagnetic particles as a carrier (matrix) for amino coupling immobilization of a bait protein [9]. Such particles provide a fast and convenient way to “catch” affinity proteins that bind to the bait protein from complex systems, such as blood serum or tissue lysates. Using a magnetic tripod or a magnetic pipette, all paramagnetic particles with a “fished” material are collected from the sample and transferred to a buffer for washing the proteins associated with the “bait”, and the paramagnetic particles can be reused.

Thus, using series of such manipulations, it is possible to extract from a biological material of interest the partner proteins to the target protein immobilized on paramagnetic particles and the amounts of the fished molecules is sufficient for MS identification or SPR analysis [11].

Initially, we tested a variant of direct molecular fishing with the use of various paramagnetic nanoparticles fluid MAG-CMX (Chemicell, Berlin, Germany) with magnet core coated by carboxymethylated dextran (size 50–200 nm). However, these experiments demonstrated an unacceptably high level of nonspecific sorption of proteins on such paramagnetic nanoparticles. Better results were obtained in the case of the use of paramagnetic microparticles of Sepharose (NHS Mag Sepharose, GE Healthcare) as a carrier [9]. These paramagnetic spherical particles coated by highly cross-linked agarose with N-hydroxysuccinimide (NHS) had the average size 1–10 µm.

Direct fishing on such Sepharose paramagnetic microparticles has been successfully used for identification in human liver tissue lysate of potential protein partners of two target proteins (microsomal cytochrome b5 and transthyretin) encoded by the genes located on human chromosome 18 [12].

## 3. Application of the SPR Technology at Various Stages of Direct Molecular Fishing

In subsequent studies we actively used the SPR technology not only at the final stage of research to validate paired interactions between the target protein and its isolated/identified protein partners, but also at other stages of direct molecular fishing [13]. These stages are marked with red numbers in Figure 1.

### 3.1. Optimization of the Protocol of Bait Protein Immobilization on a Carrier

**Figure 2:**
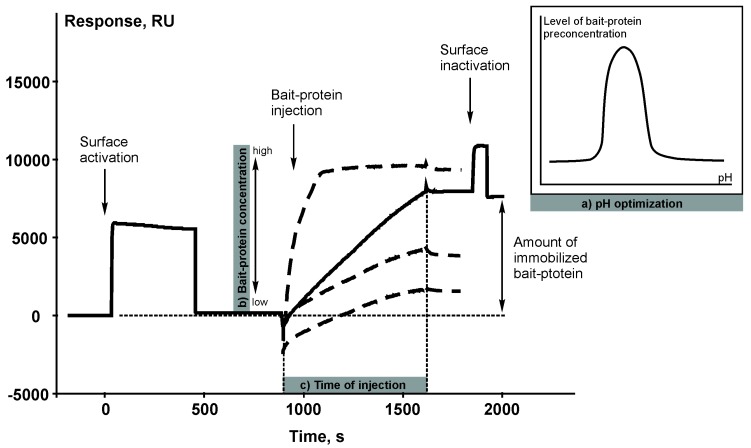
An example of the sensorgram of immobilization of a bait protein (cytochrome b5). 1 RU corresponds to 1 pg of the bound substance per 1 mm^2^ of the chip surface. The following parameters of the immobilization protocol can be optimized by using the SPR biosensor: (**a**) a pH value of the immobilization buffer; (**b**) concentrations of the bait protein (bold dashed line shows different concentrations of the bait protein); (**c**) time of injection.

The chemical reaction of covalent immobilization of the target bait protein on a carrier (sorbent or microparticles) is basically the same as immobilization of a protein ligand on the surface of a SPR chip coated with a carboxylated dextran layer. The principle of covalent immobilization is based on amide bond formation between the N-terminal amino group or amino group of lysine residues of a bait protein and carboxyl group of dextran polymer using *N*-hydroxysuccinimide/1-ethyl-3-[3-dimethylaminopropyl] carbodiimide coupling reaction. Therefore, the sensorgram of immobilization of a bait protein (Figure 2) represents a visualization of the process of creating an affinity reagent.

### 3.2. Validation of Intactness of the Immobilized Bait Protein

Immobilization of the target protein on any carrier always raises a question, whether the immobilized protein remains native after the immobilization procedure. In the case of interaction of the target bait protein with known protein partners the SPR biosensor makes it possible to control functional competence of the immobilized protein in terms of its participation in known protein–protein interactions.

**Figure 3:**
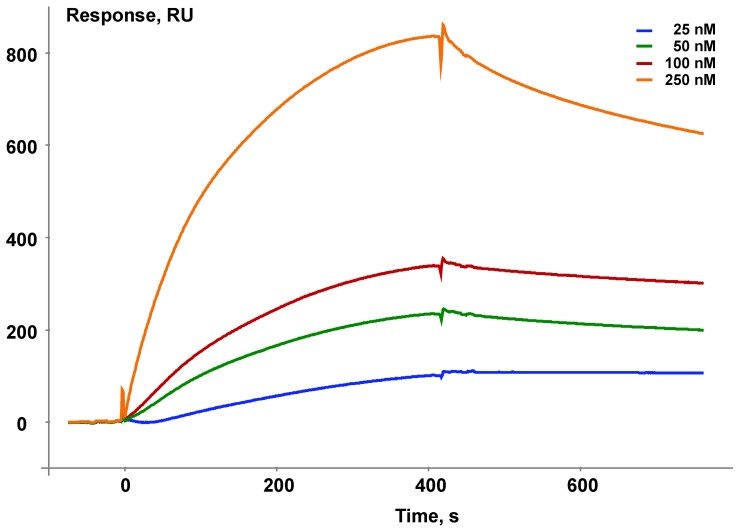
An example of a series of sensorgrams illustrating validation of nativity of the immobilized bait protein CYB5A. Different colors show different concentrations of the partner protein, CYP1B1.

As an example, Figure 3 shows validation of the ability of the immobilized bait protein microsomal cytochrome b5 (CYB5A) to interact with its known protein partner cytochrome P450 1B1 (CYP1B1).

### 3.3. Optimization of the Protocol for Preparation of Tissue/Cell Culture Lysates

**Figure 4:**
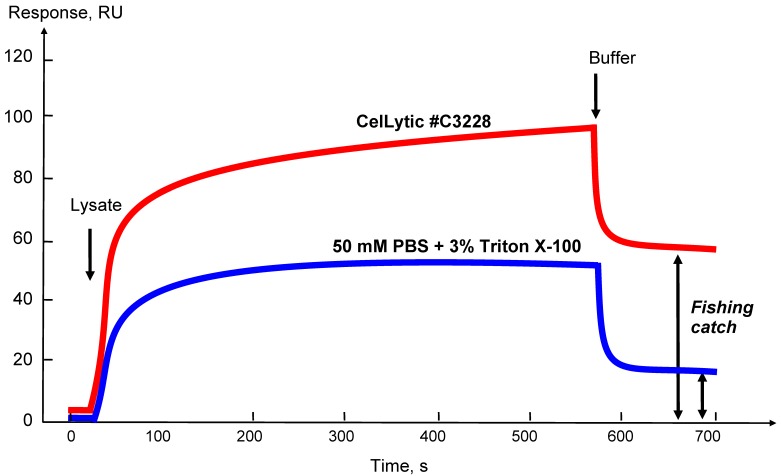
Sensorgrams of affinity molecule binding from lysate samples obtained using different lysis buffers. CelLytic—Cell Lysis Reagent for mammalian tissues (Sigma Aldrich, Cat. No. C3228, St. Louis, MO, USA). The double arrows (↕) show the residual level of the biosensor signal after injection; it reflects the level of possible fishing catch.

The choice of a lysis buffer and optimization of the protocol of lysis of certain types of the biological samples with an estimate of the amount of “fished” molecules that can be caught on a bait protein is conveniently controlled by an optical SPR biosensor. Figure 4 shows that using the biosensor sensorgram curves it is possible to estimate the extracting efficiency of affinity protein partners from the biomaterial onto the target protein in the case of the use of different lysis buffers or by varying any parameter of the lysis protocol. In addition, the level of the residual signal after injection of the sample can approximately estimate the amount of the molecular “fish” that can be caught from this lysate (as shown in Figure 4 by double arrows).

### 3.4. SPR Modeling of Direct Molecular Fishing

Direct molecular fishing is based on the affinity interaction of partner proteins present in the lysate of the biological material with the immobilized target bait protein.

**Figure 5:**
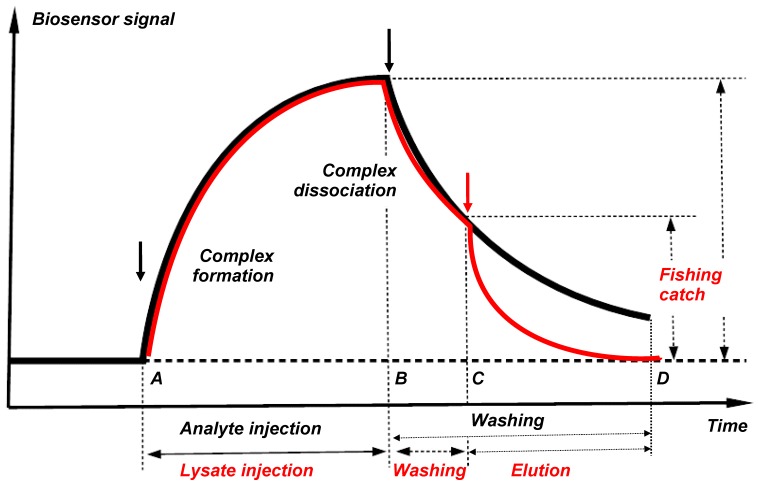
Theoretical curves for formation and dissociation of partner protein complexes with the immobilized bait protein shown in the form of a biosensor sensorgram (**black line**) and a molecular fishing curve on an affinity chromatography column (**red line**).

Figure 5 shows the theoretical curves for the formation and dissociation of protein partner complexes with the immobilized bait protein presented in the form of a biosensor sensorgram (black line) and a molecular fishing curve on an affinity chromatography column (red line). Protein complexes are formed after injection of an analyte or a biological lysate (the time interval AB). The “fished material” is then washed from non-specific impurities (the time interval B-C). In this case, a partial or total loss of associated “fished” molecules can occur; this depends on tightness of the complexes formed and duration of washing. Therefore, a sufficiently high affinity of the interacting (bait-fished) molecules is an important precondition for successful fishing; such affinity provides retention of a significant proportion of the “fished molecules” on the immobilized bait protein throughout the washing procedure. Our analysis of the SPR sensorgrams of interaction of 12 pairs of proteins with Kd values ranged from 10^−5^ M to 10^−9^ M has shown that applicability of direct molecular fishing is limited by the Kd value not higher than 10^−5^ M [14]. At Kd values higher than 10^−5^ M it is highly likely that protein partners will be readily removed from the bait during washing procedure.

This limitation has both positive and negative sides. The “fished material” should be free of include “sticky” proteins with high nonspecific sorption, since Kd values of such nonspecific interactions >10^−4^ M. The negative side is that low-affinity protein partners that form short-lived complexes with the target protein cannot be detected because of their removal during the washing procedure.

Thus, injecting the biological lysate through the biosensor channel with the immobilized target protein, we can optimize various parameters of the fishing protocol for this particular bait protein and use the optimized protocol for the preparative fishing on the affinity chromatographic column. The following parameters can be optimized: (1) lysate concentration(s) and composition of the running buffer; (2) time and a flow rate of lysate injection; (3) composition of the washing buffer and washing time; (3) the composition of the elution buffer and elution time; (4) quantitative assessment of the “fished material”.

The total volume of a chromatographic column determines evident advantages in the specific capture of protein partners while using the SPR biosensor. It is possible to detect those lysate samples or their SEC-separated fractions, which preferentially contain the protein material of interest. Evidently, the higher the concentration of protein partners is in the lysate, the higher the SPR signal reflecting specific binding to the protein immobilized on the chip. Thus, it is reasonable to use the optimized concentration of the lysate sample determined during the SPR biosensor-based analysis for column chromatography. Results of the SPR-based analysis also allow the prediction of and possibly the minimization of the impact of background proteins on specific capture of protein partners by evaluating not only specific sorption onto the immobilized protein but also non-specific sorption onto the chip surface. Thus, although the use of the SPR biosensor is optional, it is basically needed for pilot modeling of processes that do occur during preparative fishing on the affinity chromatographic column.

### 3.5. Quantitative Assessment of the “Fished Material”

Before LC-MS/MS analysis of the eluate obtained from molecular fishing on an affinity chromatographic column, performing pilot evaluation of the amount of “fished material” by means of an optical SPR biosensor containing the immobilized bait protein in one of its channels is optional. Taking into consideration that the eluate may contain components impairing PPI, it is necessary before biosensor analysis to replace the elution buffer with a filter column by a more suitable buffer. Such simple procedure results in a sensorgram reflecting the level (expressed in RU units) of affinity molecule binding from the eluate to the immobilized bait protein. Since 1 RU corresponds to 1 pg of the bound substance per 1 mm^2^ of the chip surface, we can make an approximate quantitative estimation of total amount of the “fished material”. Thus, the use of an optical SPR biosensor at this stage gives an idea about efficiency of the molecular fishing on the affinity chromatographic column, and whether the collected fished material is sufficient for subsequent LC-MS/MS protein identification.

### 3.6. Validation of Paired Protein–Protein Interactions

The list of proteins identified during LC-MS/MS analysis should be validated using an optical SPR biosensor for the presence of real partner proteins to the target protein among the identified candidates. For this purpose, available purified proteins from this list are used for direct SPR-based molecular interaction with the immobilized bait protein and affinity partner proteins are thus selected for more detailed binding analysis with the target protein. Further studies may include analysis of kinetic and thermodynamic characteristics of the PPI. This application of the optical SPR biosensor is classical and it reveals its main purpose: analysis of intermolecular interactions and determination of the most important kinetic and thermodynamic characteristics of such interactions. As an example, Table 1 provides a list of earlier validated paired protein–protein interactions.

## 4. SPR-Based Analytical Fishing

**Figure 6:**
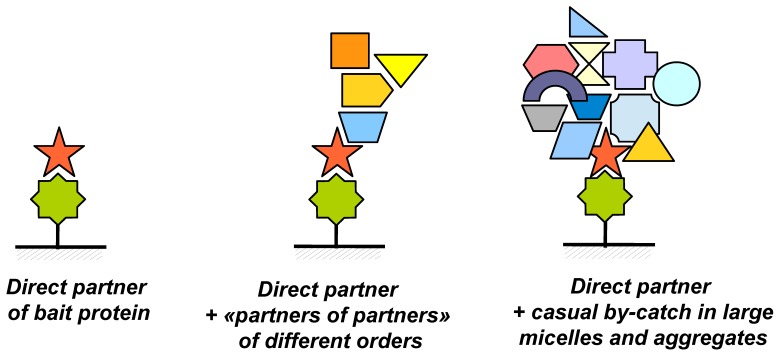
Illustration of the possible causes of appearance of a large number of proteins in the “fished material” after fishing: (**A**)—partners of the bait protein, (**B**)—partners of different orders (“partners of partners”), (**C**)—random “neighbors” in nonspecific complexes and micelles.

The practice of direct molecular fishing of proteins from the whole tissue lysate (liver tissue lysate as an example) has shown that from dozens to hundreds of proteins can be fished using a target bait protein [12,13]. In addition, the protein composition of the “fished material” for each bait protein is quite specific—lists of identified proteins coincide only by 2–5%. This result is explained by contaminations with proteins, which are indirectly related to the studied affinity interactions. This problem is illustrated in Figure 6. During affinity isolation, the immobilized bait protein may interact not only with single specific protein partners (Figure 6A) but also with larger complexes in which the partner protein plays a role of a tag, which promotes fishing of such complexes (Figure 6B). Other components present in such complexes may not interact directly with the bait protein and therefore they may be defined as higher order partners (“partners of partners”); such proteins having indirect functional links with the immobilized target protein.

The fished material may also contain various micelles and aggregates (Figure 6C) also labeled by real protein partners. In such objects there can be absolutely “foreign” proteins entered such supramolecular particles during homogenization and lysis of the initial biological material. Obviously, this problem may be solved by means of validation of potential partner proteins by using highly purified protein preparations and the SPR biosensor technology.

To minimize nonspecific interactions, we have developed a method for targeted depletion/enrichment of the lysate, based on combination of size-exclusion chromatographic (SEC) fractionation of the analyzed lysate with analytical fishing in the SPR biosensor.

**Figure 7:**
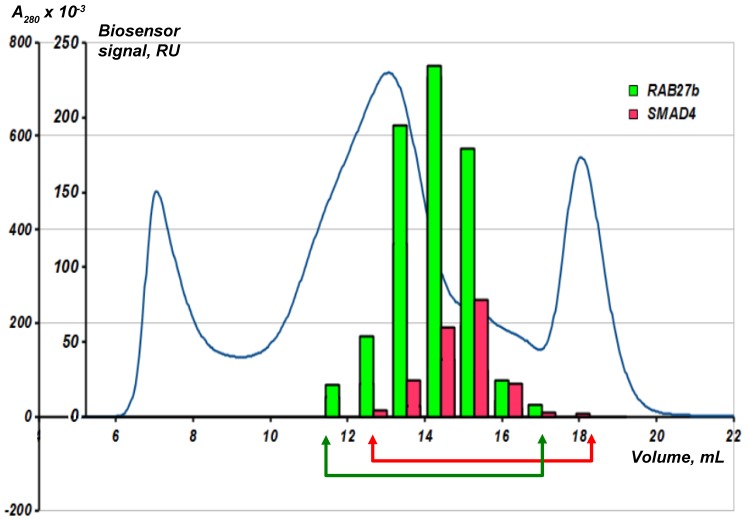
Combination of SEC fractionation of the biological lysate with analytical fishing on the SPR biosensor chip.

Fractions obtained after the gel-chromatographic lysate separation are collected in a standard 96-well plate and transferred to the SPR biosensor. All the fractions are sequentially analyzed in the automatic mode for the presence of molecular objects (“fished material”) interacting with the bait protein immobilized on an optical chip. Figure 7 shows an example of such an analysis using two immobilized bait proteins RAB27b and SMAD4.

The blue line is the optical density at 280 nm. The human liver tissue lysate was fractionated on a Superose 6 column. Analytical fishing was performed using the Biacore 3000 SPR biosensor and an optical chip CM5 with the immobilized target bait proteins RAB27b and SMAD4. The arrows show an individual selection of fractions for each bait protein for the subsequent preparative fishing.

It is interesting to note that the absorbance peak in the SEC chromatogram and the peak in binding magnitude for the RAB27b by SPR are offset from each other. This may be attributed to the presence of large amounts of background proteins. The chromatogram reflects total protein content in the fraction. At the same time, the content of partner proteins may not be proportional to the content of the total protein in the fraction. During the SPR-analysis, all fractions are ranked according to the content of partner proteins for the target protein, and for the preparative fishing the fractions with the maximum protein content of the partners are used. This underlines the importance of the preliminary SEC and SPR analysis of the resultant lysate fractions before the preparative molecular fishing.

Using the analytical fishing procedure, it is possible to obtain for each bait protein specific fractions of the lysate enriched with fished proteins and depleted by contaminated proteins. These fractions are further used in preparative direct molecular fishing; such procedure results in a 5–10-fold decrease in the number of fished proteins. For example, after lysate pre-fractionation the number of fished proteins bound to the immobilized bait protein CYB5A decreased from 98 to 18 [13].

## 5. Direct Molecular Fishing Using Non-Peptide Small Molecule Ligands: Focus on Isatin

All the above considered strategies are basically applicable for molecular fishing using non-peptide small molecules as ligands. Below we demonstrate one such example of the use of a small molecule for direct molecular fishing and studies of protein–protein interactions.

Isatin (indole-2,3-dione) is an endogenous indole found in the mammalian brain, peripheral tissues, and biological fluids [16]. Isatin analogs exhibit various pharmacological activities, which are determined by the chemical structure of substituents introduced in the indole nucleus. The isatin moiety is often present in various pharmacologically attractive compounds exhibiting properties of inhibitors of apoptosis, anticonvulsants, antiviral, antibacterial, antifungal agents, etc. [17,18,19].

However, intact isatin molecule could not be used as an affinity ligand because both oxo groups of isatin are essential for manifestation of its biological activity [20,21]. Thus, immobilization of isatin as the bait molecule requires modification of its structure. Currently, two isatin derivatives have been used as affinity ligands for fishing of isatin-binding proteins.

The choice of a 5-amino-substituted isatin analogue was determined by the following reasons: (i) 5-aminoisatin has been successfully used for development of the ELISA method for determination of isatin in urine [22]; (ii) an amino group is suitable for immobilization; (iii) the most sensitive biological target of isatin, receptor guanylyl cyclase stimulated by atrial natriuretic peptide demonstrated even higher sensitivity to inhibition by 5-aminoisatin [21]. Using 5-aminoisatin immobilized on an SPR optical biosensor chip, it was possible to characterize in the first approximation isatin-binding activity of rat tissues [23].

Analysis of isatin-binding activity in rat tissues showed an uneven distribution of isatin binding sites between the soluble and membrane fractions of the studied organs [23]. In the membrane fraction tissue isatin-binding decreased in the following order: brainstem > hemispheres = cerebellum > heart > kidney > liver. In the soluble fraction the distribution of isatin binding decreased in the following order: kidney > heart > brainstem = hemispheres > liver > cerebellum. Investigated organs and tissues differed in form of kinetic curves of interaction with immobilized isatin analogue. The brain isatin-binding activity was also detected using as a “longer” isatin analogue, *N*-(6-aminocaproyl)-5-aminoisatin as a ligand immobilized on the SPR biosensor chip. Subsequent proteomic analysis of isatin-binding brain proteins isolated by affinity chromatography on 5-aminoisatin-Sepharose and 5-aminocaproylisatin Sepharose [7,24] showed, that in the case of a shorter isatin analogue (5-aminoisatin) the number of identified proteins was higher than using 5-aminocaproylisatin (88 vs. 66), and only 22 of protein were common to both proteomic profiles. This suggests that the length of the insert between the amino group used for covalent immobilization of the affinity ligand to Sepharose and isatin itself influences proteomic profiling of brain isatin-binding proteins. The experimental testing of this hypothesis by means of both isatin analogues immobilized on the SPR biosensor chip and several highly purified proteins revealed different affinity for these proteins to the immobilized analogues (Table 2).

SPR biosensor-based studies of protein–protein interactions revealed an interesting fact that created a new direction in the isatin research. SPR experiments have shown that isatin can regulate protein–protein interactions: in some cases, it promotes complex dissociation, while in other cases it favors complex formation.

**Figure 8:**
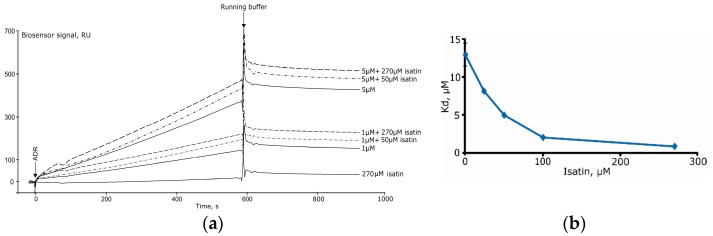
(**a**) The effect of isatin on interaction of immobilized human ferrochelatase with NADPH-dependent adrenodoxin reductase (ADR) and (**b**) the Kd values for the complex formation between these proteins (modified from [15]).

Isatin significantly increased Kd values for complexes formed by amyloid-β peptide and glyceraldehyde-3-phosphate dehydrogenase [8]. In the case of complex formation between ferrochelatase and NADPH-dependent adrenodoxin reductase [15] increasing concentrations of isatin (25–250 μM) significantly decreased Kd (Figure 8A,B) [15]. It is especially interesting that isatin poorly interacts with each individual protein separately so that isatin binding capacity of either protein cannot account for increased complex formation [15].

All these data suggest that isatin exhibits dual effects on formation of various protein complexes. It appears that interacting with a wide range of biological targets isatin acts on particular subinteractomes of the cells rather than on selected protein targets.

Elucidation of these dual effects on PPI requires an extended use of the SPR-based analysis, which should be aimed at (i) validation of isatin-binding capacity in the extended list of identified proteins fished from the biological material onto an immobilized isatin analogue as a bait; (ii) elucidation of Kd values for interacting pairs of proteins in the absence and the presence of various isatin concentrations. Using known bioinformatic data on functioning of the identified proteins as hubs in various metabolic/regulatory pathways it will be possible to create comprehensive isatin-sensitive subinteractomes. The final validation would include combined analysis and isatin-binding capacity the effect of isatin on PPI from different subinteractomes. This step clearly needs the used of the SPR-based technology again.

## 6. Conclusions

We have developed an original experimental approach employing SPR biosensors and applicable for investigation of potential partners involved in protein–protein as well as protein–peptide or protein–small molecule interactions. This approach is based on combination of the SPR technology, size-exclusion chromatography (SEC), direct molecular fishing and mass spectrometric proteins identification. It makes it possible to find and isolate specific partner proteins from the tissue/cell culture lysates by direct molecular fishing onto target protein or small molecule as baits.

In such studies the SPR technology can be actively used not only at the final stage of research for validation paired interactions between the target bait molecule and its isolated/identified protein partners, but also at other stages of direct molecular fishing. Particularly, the SPR technology allows the modeling, optimization and control of the following steps: (1) immobilization parameters (pH of the immobilization buffer, concentrations of the bait protein, time of protein sample injection); (2) validation of intactness of the immobilized bait protein; (3) comparative efficiency of different lysis buffers during the preparation of tissue/cell culture lysates; (4) suitable lysate concentration and composition of the running buffer, time and a flow rate of lysate injection, composition of the washing buffer and washing time, composition of the elution buffer and elution time; (5) quantitative assessment of the “fished material” in eluate; (6) validation of paired interactions between the target bait protein (or small compound) and “fished proteins” with determination of kinetic rate constants and equilibrium dissociation constant (Kd); (7) the analytical fishing procedure enables characterizing SEC fractions of the lysate enriched with partner proteins for any target bait protein.

Thus, in this review we have demonstrated that logic combination of SPR biosensor with several other methods makes possible the deduction of protein interactomics to a new level that brings us closer to solving the problems of functional proteomics.

## Figures and Tables

**Table 1:** The list of validated protein–protein interactions (PPI) among “fished” protein partners ^1^ of human cytochrome b5 (CYB5A) and ferrochelatase (FECH) as baits by SPR analysis.

PPI	Kd (Mean Values), M	Reference
CYB5A/CYP1B1 ^1^	7.0 × 10^−8^	[12]
CYB5A/CYP2C9	1.1 × 10^−6^	[12]
CYB5A/GAPDH	1.0 × 10^−7^	[12]
FECH/ADR	1.3 × 10^−5^	[15]
FECH/SMAD4	1.6 × 10^−6^	[15]
FECH/CYB5A	1.5 × 10^−6^	[15]

^1^ CYP1B1 and CYP2C9—isoenzymes of the cytochrome P450 family, GAPDH—glyceraldehyde-3-phosphate dehydrogenase, ADR—NADPH-adrenodoxin reductase, SMAD4—member 4 of the SMAD family of signal transduction proteins.

**Table 2:** Quantitative interaction of purified proteins with isatin analogues immobilized on the Biacore optical biosensor chip (modified from [16]).

Protein	5-aminoisatin Kd, M	5-aminocaproylisatin Kd, M
Rabbit GAPDH intact	(2.2 ± 0.6) × 10^−6^	-
Rabbit GAPDH oxidized	(8.9 ± 1.9) × 10^−6^	-
Human recombinant cytokeratin 14	ND	(7 ± 5) × 10^−7^
Human recombinant cytokeratin 8	(6.1 ± 0.3) × 10^−7^	ND
Human recombinant peroxiredoxin	(8.8 ± 0.4) × 10^−8^	(5.3 ± 0.2) × 10^−7^
Rabbit creatine phosphokinase	(2.9 ± 0.4) × 10^−7^	(7.7 ± 3.1) × 10^−8^
Rabbit glycogen phosphorylase	(3.0 ± 0.6) × 10^−5^	(4.6 ± 3.8) × 10^−5^

## References

[1] Ngounou Wetie A.G., Sokolowska I., Woods A.G., Roy U., Deinhardt K., Darie C.C. (2014). Protein-protein interactions: Switch from classical methods to proteomics and bioinformatics-based approaches. Cell. Mol. Life Sci..

[2] Braun P., Gingras A.-C. (2012). History of protein-protein interactions: From egg-white to complex networks. Proteomics.

[3] Sprinzak E., Sattath S., Margalit H. (2003). How reliable are experimental protein-protein interaction data?. J. Mol. Biol..

[4] Ivanov A.S., Zgoda V.G., Archakov A.I. (2011). Technologies of protein interactomics: A review. Russ. J. Bioorganic Chem..

[5] Tate S., Larsen B., Bonner R., Gingras A.-C. (2013). Label-free quantitative proteomics trends for protein-protein interactions. J. Proteom..

[6] Medvedev A., Kopylov A., Buneeva O., Zgoda V., Archakov A. (2012). Affinity-based proteomic profiling: Problems and achievements. Proteomics.

[7] Buneeva O., Gnedenko O., Zgoda V., Kopylov A., Glover V., Ivanov A., Medvedev A., Archakov A. (2010). Isatin-binding proteins of rat and mouse brain: Proteomic identification and optical biosensor validation. Proteomics.

[8] Medvedev A.E., Buneeva O.A., Kopylov A.T., Gnedenko O.V., Medvedeva M.V., Kozin S.A., Ivanov A.S., Zgoda V.G., Makarov A.A. (2014). The effects of endogenous non-peptide molecule isatin and hydrogen peroxide on proteomic profiling of rat brain amyloid-β binding proteins: Relevance to Alzheimer’s disease?. Int. J. Mol. Sci..

[9] Ershov P., Mezentsev Y., Gnedenko O., Mukha D., Yantsevich A., Britikov V., Kaluzhskiy L., Yablokov E., Molnar A., Ivanov A. (2012). Protein interactomics based on direct molecular fishing on paramagnetic particles: Experimental simulation and SPR validation. Proteomics.

[10] Ivanov A.S., Ershov P.V., Mezentsev Y.V., Poverennaya E.V., Lisitsa A.V., Archakov A.I. (2012). Protocols of protein interactomics: Molecular fishing on optical chips and magnetic nanoparticles. Biochem. Mosc. Suppl. Ser. B Biomed. Chem..

[11] Franzreb M., Siemann-Herzberg M., Hobley T.J., Thomas O.R.T. (2006). Protein purification using magnetic adsorbent particles. Appl. Microbiol. Biotechnol..

[12] Ivanov A.S., Medvedev A., Ershov P., Molnar A., Mezentsev Y., Yablokov E., Kaluzhsky L., Gnedenko O., Buneeva O., Haidukevich I. (2014). Protein interactomics based on direct molecular fishing on paramagnetic particles: Practical realization and further SPR validation. Proteomics.

[13] Ivanov A.S., Ershov P.V., Molnar A.A., Mezentsev Y.V., Kaluzhskiy L.A., Yablokov E.O., Florinskaya A.V., Gnedenko O.V., Medvedev A.E., Kozin S.A. (2016). Direct molecular fishing in molecular partners investigation in protein–protein and protein–peptide interactions. Russ. J. Bioorg. Chem..

[14] Ivanov A.S., Medvedev A.E. (2016). Optical surface plasmon resonance biosensors in molecular fishing. Biochem. Mosc. Suppl. Ser. B Biomed. Chem..

[15] Ershov P., Mezentsev Y., Gilep A., Usanov S., Buneeva O., Medvedev A., Ivanov A. (2017). Isatin-induced increase in the affinity of human ferrochelatase and adrenodoxin reductase interaction. Protein Sci..

[16] Medvedev A., Buneeva O., Gnedenko O., Ershov P., Ivanov A. (2018). Isatin, an endogenous nonpeptide biofactor: A review of its molecular targets, mechanisms of actions, and their biomedical implications. Biofactors.

[17] Medvedev A., Buneeva O., Glover V. (2007). Biological targets for isatin and its analogues: Implications for therapy. Biologics.

[18] Pandeya S.N., Smitha S., Jyoti M., Sridhar S.K. (2005). Biological activities of isatin and its derivatives. Acta Pharm..

[19] Limpachayaporn P., Schäfers M., Haufe G. (2015). Isatin sulfonamides: Potent caspases-3 and -7 inhibitors, and promising PET and SPECT radiotracers for apoptosis imaging. Future Med. Chem..

[20] Medvedev A.E., Goodwin B., Clow A., Halket J., Glover V., Sandler M. (1992). Inhibitory potency of some isatin analogues on human monoamine oxidase A and B. Biochem. Pharmacol..

[21] Medvedev A.E., Goodwin B.L., Sandler M., Glover V. (1999). Efficacy of isatin analogues as antagonists of rat brain and heart atrial natriuretic peptide receptors coupled to particulate guanylyl cyclase. Biochem. Pharmacol..

[22] Pang F.-Y., Hucklebridge F.H., Forster G., Tan K., Clow A. (1998). The relationship between isatin and monoamine oxidase-B inhibitory activity in urine. Stress Med..

[23] Ivanov I.D., Panova N.G., Gnedenko O.V., Buneeva O.A., Medvedev A.E., Archakov A.I. (2002). Study of the tissue and subcellular distribution of isatin-binding proteins with optical biosensor. Vopr. Med. Khim..

[24] Buneeva O.A., Kopylov A.T., Tikhonova O.V., Zgoda V.G., Medvedev A.E., Archakov A.I. (2012). Effect of affinity Sorbent on proteomic profiling of isatin-binding proteins of mouse brain. Biochemistry.

